# TAK1 deficiency in dendritic cells inhibits adaptive immunity in SRBC‐immunized C57BL/6 mice

**DOI:** 10.1002/2211-5463.12062

**Published:** 2016-04-21

**Authors:** Yao Pan, Zhiming Lei, Xuetao Wei, Weidong Hao

**Affiliations:** ^1^ Department of ToxicologySchool of Public HealthPeking UniversityBeijingChina; ^2^ Beijing Key Laboratory of Toxicological Research and Risk Assessment for Food SafetyBeijingChina

**Keywords:** dendritic cells, sheep red blood cells, T‐cell dependent antigen response, transforming growth factor‐β‐activated kinase 1

## Abstract

Dendritic cells (DCs) are important in the initiation of primary T‐cell responses, while transforming growth factor‐β (TGF‐β)‐activated kinase 1 (TAK1) is a critical regulator of DC survival and homeostasis. This study evaluated the T‐cell dependent antibody response (TDAR) to sheep red blood cells (SRBC) on a DC‐specific TAK1‐deficient mice model. The results showed that TAK1 deficiency in DCs significantly suppressed the humoral and cellular immune response in mice. DC‐specific TAK1 deletion impaired splenic T‐cell population and conventional DCs, abolished the cytokine production of splenic T cells and down‐regulated some functional gene expression in the spleen. Collectively, this study suggests that TAK1 plays an essential role in the development of the humoral immune response.

**Abbreviations**
APC: antigen‐presenting cell
DC: dendritic cell
DTH: delayed type hypersensitivity
IFN: interferon
IL: interleukin
MHC: major histocompatibility complex
PFC: plaque‐forming cell
SRBC: sheep red blood cells
TAK1: transforming growth factor‐β activated kinase 1
TDAR: T‐cell dependent antibody response
Th cell: T‐helper cells
WT: wild‐type

Dendritic cells (DCs), a morphologically distinct form of antigen‐presenting cell (APC), can capture, process, and present antigens in the form of peptides bound to major histocompatibility complex (MHC) molecules to activate T cells. It has been reported that *in vivo* antigen targeting to DCs induces strong T‐cell priming and long‐lived T cell help for antibody response 1, 2. DCs are famous for their role in activating and expanding T‐helper (Th) cells, which in turn induce B‐cell growth and immunoglobulin secretion 3. Immunoglobulin class‐switching also requires interaction between B cells and DCs 4. Therefore, DCs play a central role in initiating and modulating humoral immunity.

Transforming growth factor‐β (TGF‐β)‐activated kinase 1 (TAK1, encoded by *Map3k7*) belongs to the mitogen‐activated protein kinase kinase kinase (MAP3K) family 5. As a key regulator in inflammatory and immune signaling pathway, it can be activated by various stimuli 6, 7. TAK1 has an essential effect on promoting the survival, proliferation and function of adaptive immune cells T and B lymphocytes 6, 8, 9, 10, 11. Besides, it also controls the homeostasis of innate immune cells such as DCs 12, NK cells 13 and neutrophils 14, 15. Sato *et al*. 6 reported that antigen‐induced humoral immune responses to T‐cell dependent and independent antigens were impaired in B cell‐specific TAK1‐deficient mice. Wang *et al*. 12 showed that deficiency of TAK1 in DCs caused apoptosis and depletion of DC subsets in lymphoid and nonlymphoid tissues and suppressed T‐cell priming. But how DC‐specific TAK1 deficiency affects T‐cell dependent antibody response (TDAR) has not been reported.

The TDAR depends on the cooperation and interaction of numerous immune cell types, including APCs, such as DCs, Th cells and B cells 16. The TDAR is used as an immunotoxicity index, because it represents a comprehensive evaluation of immune function based on the assessment of various components of the immune system. Sheep red blood cell (SRBC) is a common T‐cell dependent antigen to evaluate immune status in mice. After immunized with SRBC, this SRBC‐specific IgM response in spleen can be measured by plaque‐forming cell (PFC) assay 17. This test is an IgM/complement dependent *in vitro* assay in mice, with SRBC as both the antigen and the target for complement mediated lysis. The SRBC PFC assay is considered the ‘gold standard’ for TDAR based on extensive intra‐ and inter‐laboratory validation in mice and the fact that it has been utilized for over 35 years 18. Although PFC assay is commonly used for assessing the potential immunotoxicity of xenobiotics 19, 20, 21, we used it in this study to evaluate the function of TAK1 in DCs on humoral immune response due to its integrated assessment capability and its sensitivity and stability.

The purpose of the study was to investigate the effect of DC‐specific TAK1 deficiency on adaptive immune response in SRBC‐immunized mice and to identify the impact of TAK1 in DCs on maintaining immune homeostasis and function. Here, we immunized the animals with SRBC and then performed functional assays including PFC assay, hemolysis test, and delayed type hypersensitivity (DTH) and quantified the antibody subsets in serum. Furthermore, splenic immune cell subpopulations, splenic T‐cell cytokine production and splenic functional gene expressions were also detected.

## Materials and methods

### Experimental animals

Floxed *Map3k7* (*Map3k7* ^fl/fl^) 8 and CD11c‐Cre 22 mice were purchased from the Jackson Laboratory (Bar Harbor, ME, USA). All mice are on C57BL/6 background. To delete TAK1 specifically in CD11c^+^DCs, we crossed mice bearing loxP‐flanked alleles of the *Map3k7* gene with mice expressing Cre under the control of the CD11c promoter to generate *Map3k7* ^fl/fl^ CD11c‐Cre mice (called ‘*Map3k7* ^DC^ mice’ hereafter). Wild‐type (WT) controls were in the same genetic background and included Cre+ mice to account for Cre effects. Guinea pigs (250 g) were used for the preparation of complement for PFC assay and hemolysis test and they were purchased from Vital River Laboratory Animal Technology Co. Ltd (Beijing, China). All animals were healthy, housed in a barrier system (temperature: 20–26 °C; relative humidity: 40–70%) with a 12 h light/dark cycle. Water and standard diet were available *ad libitum*. The study was approved by the Animal Experimental Welfare & Ethical Inspection Committee of Peking University. Animal experiments and housing procedures were carried out in accordance with the laboratory animal administration rules of the Ministry of Science and Technology of the People's Republic of China.

### Chemicals and antibodies

Sheep red blood cells (SRBC) were purchased from Vital River Laboratory Animal Technology Co. Ltd. PE‐Cy^™^5 hamster anti‐mouse CD3e, PE rat anti‐mouse CD8a, FITC hamster anti‐mouse CD11c and PE rat anti‐mouse I‐A/I‐E were purchased from BD Pharmingen (San Diego, CA, USA). Anti‐mouse CD4FITC and anti‐human/mouse CD45R (B220) PE were obtained from eBioscience (San Diego, CA, USA). Mouse IgG, IgA, and IgM ELISA kits were purchased from Freemore Biotechnology Co. Ltd (Beijing, China). Guinea pig complement, Hanks balanced salt buffer (HBSS), SA buffer solution and Drabkin's reagent were homemade.

### Preparation of spleen cell suspensions

Each spleen isolated above was transferred to the 70 μm cell strainer in culture dishes containing 4 mL of cold HBSS and smashed the tissue with the plunger of a 1 mL syringe. Filtered the splenocyte suspensions through the two‐layer carbasus on top of the tubes and washed twice in HBSS and centrifuged for 10 min at 230 ***g*** at room temperature. Splenocytes were resuspended in 10 mL HBSS. Cell numbers were determined for each splenocyte suspension by counting in a hemocytometer.

### Plaque‐forming cell assay

The Cunningham modification of Jerne and Nordin antibody plaque‐forming cell assay was used 23, 24, 25 to determine IgM production by spleen cells. Mice were immunized on Day 1 with 0.2 mL of 2% (v/v) SRBC suspension in sterile saline via intraperitoneal injection. On day 5, the mice were killed and spleen cell suspensions were prepared as stated in the part of “Preparation of spleen cell suspension”. About 20 μL of spleen cell suspension in HBSS, 50 μL of 10% (v/v) SRBC in SA buffer solution and 500 μL of agar solution (0.5 g/100 ml in HBSS, pH 7.2–7.4) were mixed in a glass tube, and then poured onto slides. The slides were inverted on a special frame after the mixtures were solidified, and incubated at 37 °C for 1.5 h, then diluted guinea pig complement (1 : 10 diluted with SA buffer solution) was added to the slot between the slides and the bottom of the frame. The slides were incubated at 37 °C for another 1.5 h, then plaque production was enumerated and the results were expressed as the number of PFC per 10^6^ splenocytes.

### Hemolysis test

Mice were immunized with SRBC as stated in the PFC assay section. The sera were obtained and assayed for HC_50_. One millilitre of SA buffer solution, 0.5 mL of 15% (v/v) SRBC, 1 mL of diluted guinea pig complement (1 : 10 diluted with SA buffer solution) and 3.3 μL of WT mice serum or 50 μL of *Map3k7* ^DC^ mice serum were added into sample tubes and the blank control was the tube without serum. All tubes were kept in water bath for 15 min at 37 °C, and then the tubes were kept in ice bath to terminate the reaction. After centrifuged for 10 min at 890 ***g***, 1 mL of supernatant was collected and added into 3 mL of Drabkin's reagent, at the same time, 0.25 mL of 15% (v/v) SRBC and 3.75 mL of Drabkin's reagent were mixed and placed for 10 min as positive control. The absorbance at 540 nm was detected using UV‐2100 spectrophotometer (UNICO Instrument Co. Ltd, Shanghai, China), the HC_50_ value was got by the equation: HC_50_ = (OD_sample_/OD_positive control_) × dilution factor (300 for WT mice and 20 for *Map3k7* ^DC^ mice).

### Serum immunoglobulin isotypes quantification

The animals were immunized with SRBC as stated in the PFC assay section. Blood for immunoglobulin isotypes quantification was collected into tubes without anticoagulant and centrifuged to obtain serum. Total IgG, IgM, and IgA titers in the serum of each animal were determined by ELISA kits. The assay procedures were in accordance with the descriptions in the manufacturer's instructions. The absorbance (450 nm) of the contents of each well was determined using FLUOstar Omega multidetection micro‐plate reader (BMG Labtech, Ortenberg, Germany).

### Delayed‐type hypersensitivity to SRBC

Mice were sensitized on Day 1 via intraperitoneal injection of 0.2 mL of 2% (v/v) SRBC in sterile saline. Five days later, the thickness of left rear footpad of each mouse was determined with a digital caliper (Mahr, Göttingen, Germany). Then, the left rear footpad was injected with 20 μL of 20% (v/v) SRBC by subcutaneous injection. After 24 h, the thickness of left rear footpad was measured again. Swelling was expressed by the difference between two measurements before and after injection of SRBC to left rear footpad.

### Flow cytometric analysis

The animals were immunized with SRBC as stated in the PFC assay section and the spleen cell suspensions were prepared as stated in the part of “Preparation of spleen cell suspension”. The splenocytes were stained with combination of antibodies in 100 μL staining buffer (2% FBS in PBS) at 4 °C for 30 min in the dark. Excessive unreacted antibody was removed by washing the cells with staining buffer. Cells were resuspended at 10^6^ cells per tube in 300 μL staining buffer and then analyzed on a FC500‐MPL flow cytometry (Beckman Coulter, Brea, CA, USA). A minimum of 20 000 events per sample was collected and analyzed for antigen expression.

### Stimulation of cytokine production and intracellular cytokine staining

The animals were immunized with SRBC as stated in the PFC assay section and the spleen cell suspensions were prepared as stated in the part of “Preparation of spleen cell suspension”. The aliquots of 2 × 10^6^ splenocytes were stimulated with cell stimulation cocktail (500×; eBioscience) for 4 h in the presence of Brefeldin A (1000×; eBiscience). The cells were stimulated in RPMI 1640 medium (Gibco, Shanghai, China) supplemented with 10% fetal bovine serum, 1% penicillin/streptomycin, 2 mm l‐glutamine and 50 μm 2‐mercaptoethanol and incubated under a humidified atmosphere of 5% CO_2_/95% air at 37 °C. Different cell surface markers and intracellular cytokines were stained with cocktails of fluorescence‐conjugated monoclonal antibodies according to the manufacturer's protocols (eBioscience).

### Gene expression in spleen

The animals were immunized with SRBC as stated in the PFC assay section. Total RNA was extracted from 1 × 10^7^ splenocytes using TRIzol reagent (Transgen Biotech, Beijing, China) according to the manufacturer's instruction. Total RNA (1 μg) was reverse transcribed with Oligo dT primer to cDNA using PrimeScript^™^ RT‐PCR kit (Takara, Dalian, China). All real‐time PCR primers were designed and synthesized by AuGCT DNA‐SYN Biotechnology (Beijing, China). Real‐time PCR primer sequences used are listed in Table 1. All real‐time PCR reactions were carried out using SYBR^®^ Premix Ex Taq^™^ II (TliRNaseH Plus) (Takara) and the IQ5 iCycler (Bio‐Rad Laboratories, Hercules, CA, USA) according to manufacturer protocols. The relative gene expression was calculated using the delta‐delta Ct (threshold cycle) method based on normalization to the reference gene *Gapdh* (glyceraldehyde‐3‐phosphate dehydrogenase).

**Table 1:** Primer sequences used for real‐time PCR

Gene	Primer sequences (5′ to 3′)	Tm (°C)
*Ifng*		
Sense	CTC AAG TGG CAT AGA TGT GG	60.0
Antisense	CAA TGA CGC TTA TGT TGT TG	56.0
*Il2*		
Sense	CTC TGC GGC ATG TTC TGG ATT	60.3
Antisense	CAG AAA GTC CAC CAC AGT TGC T	60.3
*Il4*		
Sense	GGT CTC AAC CCC CAG CTA GT	64.0
Antisense	CCC TTC TCC TGT GAC CTC GT	64.0
*Il10*		
Sense	TGGACAACATACTGCTAACC	58.0
Antisense	GGATCATTTCCGATAAGGCT	58.0
*Tbet*		
Sense	CACTAAGCAAGGACGGCGAA	62.0
Antisense	CCACCAAGACCACATCCACA	62.0
*Gata3*		
Sense	AAGAAAGGCATGAAGGACGC	60.0
Antisense	GTGTGCCCATTTGGACATCA	60.0
*Cxcr4*		
Sense	ATCCCAGCCCTCCTCCTGACTA	64.3
Antisense	TCCACAGGCTATCGGGGTAAAG	62.3
*Ccr7*		
Sense	ATGTATTCTGTCATCTGCTTCGTGG	61.3
Antisense	GCCAGGTTGAGCAGGTAGGTATC	64.3
*Mhc I*		
Sense	GCCCAAGAAGTGGATTACGGAG	62.3
Antisense	TCCAGAACCATTTGGCGACC	62.0
*Mhc II*		
Sense	CTGTCTGGATGCTTCCTGAGTTT	60.3
Antisense	CAGCTATGTTTTGCAGTCCACC	60.3
*Gapdh*		
Sense	GCT GAG TAT GTC GTG GAG T	58.0
Antisense	GTT CAC ACC CAT CAC AAA C	56.0

### Statistical analysis

Statistical analysis was performed using graphpad prism software (GraphPad, La Jolla, CA, USA). Data are expressed as mean ± standard deviation (SD). *P* values were calculated using Student's *t*‐test. Statistics with *P* value less than 0.05 were considered significant.

## Results

### The effect of TAK1 deficiency in DCs on adaptive immune response in SRBC‐immunized mice

**Figure 1:**
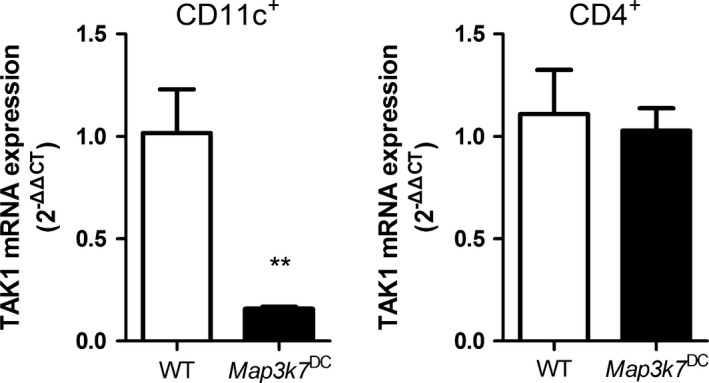
Deletion of TAK1 in DCs from *Map3k7* ^DC^ mice. Analysis of TAK1 mRNA expression in splenic DCs (CD11c^+^) and CD4^+^ T cells of WT and *Map3k7* ^DC^ mice. Data are representative of three independent experiments. The bars represented mean ± SD (*n* = 6). **P < 0.01 indicate significant changes compared to the WT group.

**Figure 2:**
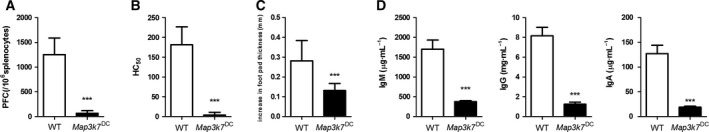
Deletion of TAK1 in DCs results in suppressed humoral and cellular immune response. WT and *Map3k7* ^DC^ mice were immunized with SRBC as stated in the PFC assay section. (A) Counts of PFCs/10^6^ splenocytes. (B) Serum hemolytic activity (HC_50_). (C) The increase in foot pad increase. (D) Determination of serum IgM, IgG, and IgA. The bars represented mean ± SD (*n* = 9). ***P < 0.001 indicate significant changes compared to the WT group.

To confirm the successful generation of *Map3k7* ^DC^ mice, we checked the TAK1 knockout efficiency in the mice. The results showed that the mRNA expression level of TAK1 was significantly decreased in splenic DCs (CD11c^+^ cells) but not CD4^+^ T cells compared to WT mice (Fig. 1). The deficiency of TAK1 in DCs induced markedly reduction in the number of lysate plaques (PFCs) counted in 10^6^ splenocytes and total serum hemolytic activity (HC_50_) (Fig. 2A,B). The deletion of TAK1 specifically in DCs elicited a suppressive effect in DTH response, as the increase in foot pad thickness was significantly reduced compared to WT controls (Fig. 2C). The serum IgA, IgM and IgG levels in *Map3k7* ^DC^ mice were dramatically lower than those of WT mice (Fig. 2D).

### The effect of DC‐specific deletion of TAK1 on spleen cell subtyping in SRBC‐immunized mice

**Figure 3:**
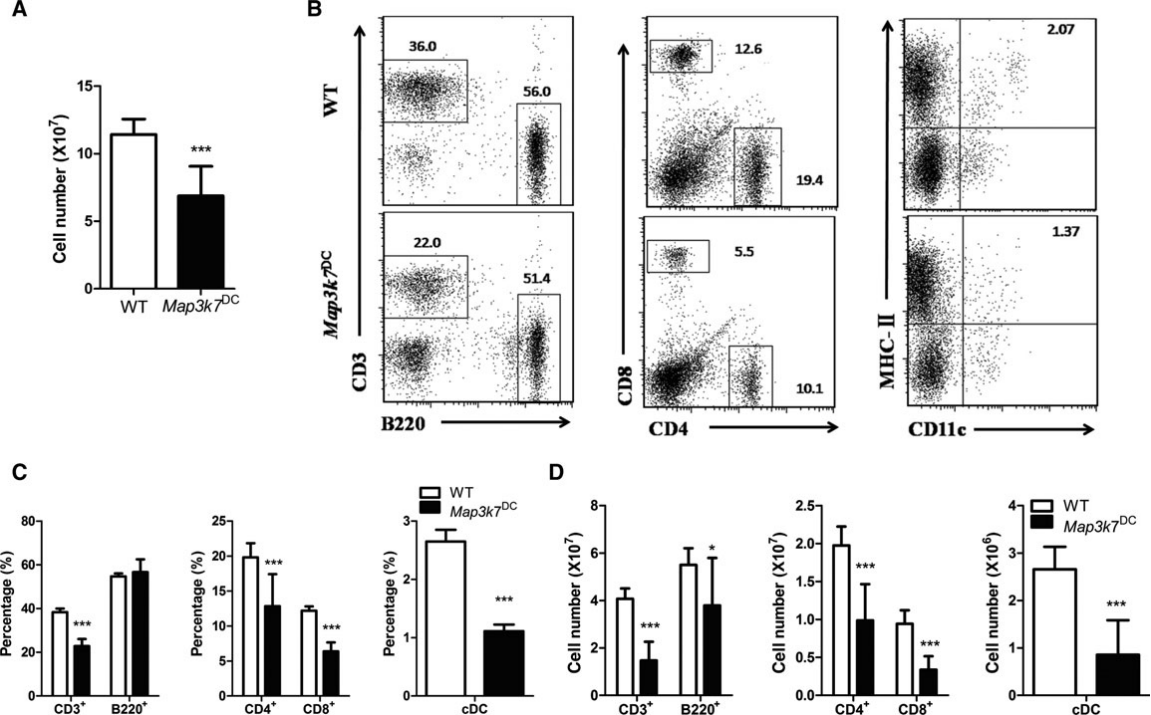
DC‐specific deficiency of TAK1 disrupts the spleen cellularity and cell subpopulation. T and *Map3k7* ^DC^ mice were immunized with SRBC as stated in the PFC assay section and the splenocytes were subtyped by flow cytometer. (A) Total cell number of spleen. (B) Flow cytometry analysis of splenic B, T, Tc, Th cells, and cDC. (C) The percentage and (D) cell numbers of splenic cell subtypes. The bars represented mean ± SD (*n* = 9).*P < 0.05, ***P < 0.001 indicate significant changes compared to the WT group.

The total number of splenocytes of *Map3k7* ^DC^ mice was significantly lower than that of WT controls (Fig. 3A). *Map3k7* ^DC^ mice had significantly reduced percentages of conventional DCs (cDCs, MHC‐II^+^CD11c^+^) and T cells (CD3^+^), especially CD8^+^ T cells (cytotoxic T cells, Tc) (Fig. 3B,C). However, there was no dramatic change in B cells (B220^+^) proportion between these two groups (Fig. 3B,C). TAK1 deficiency in DCs caused significant decrease in the counts of T cells, B cells, CD4^+^ T cells (Th cells), CD8^+^ T cells, cDCs in spleen (Fig. 3D).

### The effect of DC‐specific deficiency of TAK1 on splenocytes cytokine production in SRBC‐immunized mice

**Figure 4:**
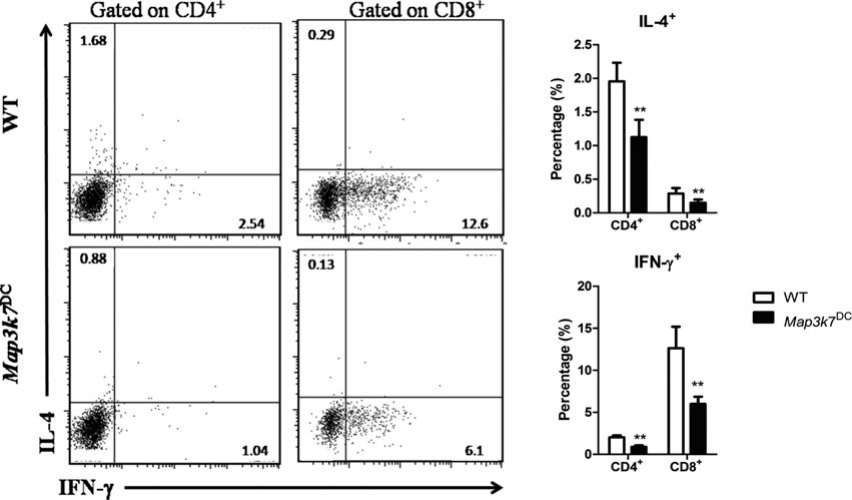
DC‐specific deletion of TAK1 impaired the cytokines production of T cells in spleen. WT and *Map3k7* ^DC^ mice were immunized with SRBC as stated in the PFC assay section and the expression of IFN‐γ and IL‐4 in splenic CD4^+^ and CD8^+^ T cells was determined by intracellular cytokine staining after stimulation with PMA and ionomycin. (Left) Flow cytometry of splenic IFN‐γ^+^ CD4^+^, IFN‐γ^+^ CD8^+^, IL‐4^+^ CD4^+^ and IL‐4^+^ CD8^+^ populations. (Right) The proportion of these populations. The bars represented mean ± SD (*n* = 6). **P < 0.01 indicate significant changes compared to the WT group.

Using intracellular cytokine staining after stimulated with PMA and ionomycin to measure the expression of cytokines in splenic T cells, we found that splenic CD4^+^ and CD8^+^ T cells from *Map3k7* ^DC^ mice secreted significantly less IFN‐γ and IL‐4 than that of WT controls (Fig. 4).

### The effect of TAK1 deletion in DCs on splenocyte gene expression in SRBC‐immunized mice

**Figure 5:**
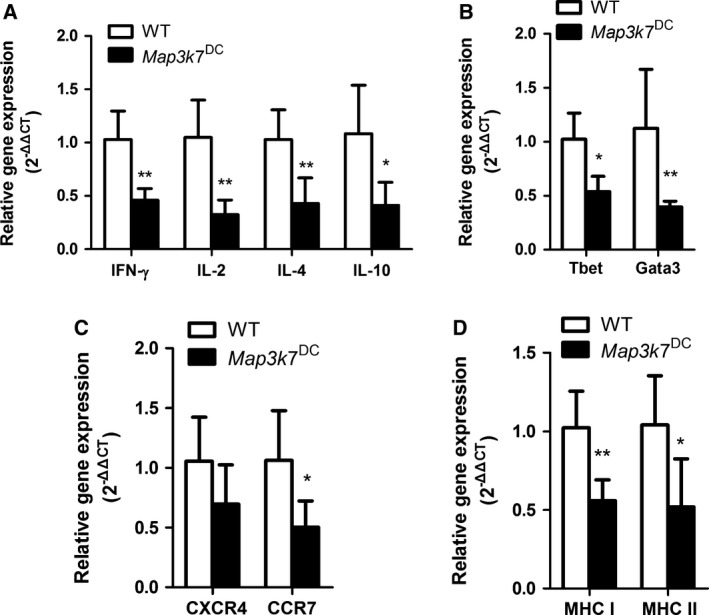
TAK1 deficiency in DCs down‐regulates the gene expression levels in spleen. WT and *Map3k7* ^DC^ mice were immunized with SRBC as stated in the PFC assay section and total RNA of spleens was analyzed for the expression levels of the indicated genes by real‐time PCR. (A) Cytokines gene expression. (B) Transcription factors gene expression. (C) Chemokine receptors gene expression. (D)MHC molecules gene expression. The bars represented mean ± SD(*n* = 6). *P < 0.05, **P < 0.01 indicate significant changes compared to the WT group.

*Map3k7* ^DC^ mice showed markedly lower expression levels of several cytokines including IFN‐γ, IL‐2, IL‐4 and IL‐10 and reduced expression of transcription factors T‐bet and GATA‐3. In addition, expression of chemokine receptor CXCR4 was slightly down‐regulated without statistical significance, but the expression of CCR7 was significantly reduced in *Map3k7* ^DC^ mice. We also detected the MHC molecules mRNA expression levels in the spleen, and both MHC‐I and MHC‐II molecules were dramatically down‐regulated in *Map3k7* ^DC^ mice (Fig. 5).

## Discussion

This study was aimed to clarify how DC‐specific TAK1‐deficient mice responded to a T‐cell dependent antigen‐SRBC and to figure out its cellular and molecular mechanism. The results of this study showed that TAK1 deficiency in DCs suppressed SRBC‐induced PFC response and DTH response and decreased the levels of HC_50_ and immunoglobulin isotypes in serum. Besides, the balance of immune cell subpopulations in spleen and the cytokines produced by splenic T cells were disrupted, while mRNA expression levels of several cytokines, transcription factors, chemokine receptor, and MHC molecules were also down‐regulated in *Map3k7* ^DC^ mice.

The adaptive immune response to the antigen includes both the cellular and humoral immunity and the mechanisms of which consist of antibody‐producing B cells, various kinds of Th cells, killer T cells and regulatory T cells. The humoral immune response induced by B cells is characterized by the specific antigen antibody reaction. The hemolysis test is an alternative to the PFC assay to measure the serum antibody level, which was first introduced in 1979 26, and it is now endorsed by Ministry of Health of China as a first‐line immune function test for evaluating the potential of health food to boost immunity 27. PFC assays is the most common method to evaluate the humoral immunity mediated by SRBC, and the amount of serum antibodies is also an accepted biomarker of the humoral immune response 28. DTH reaction is a kind of T‐cell mediated immune response to SRBC and is usually used to assess the cellular immunity in mice 29. In this study, compared with WT controls, PFC response was significantly suppressed and almost lost in *Map3k7* ^DC^ mice (Fig. 2A). A dramatically reduced level of serum HC_50_ value and immunoglobulin isotypes—IgG, IgM, and IgA—were also found in DC‐specific TAK1‐deficient mice (Fig. 2B,D). This suggests an overall effect of TAK1 in DCs on antibody production, since variations attributed to the response to the immunogen and production of specific antibody should not explain a so dramatic reduction in the global concentration of immunoglobulins. In accordance with the phenotype of other mice harboring cDC depletion 30, the antigen‐specific antibodies generation after immunization was completely abolished. DTH reaction to SRBC in *Map3k7* ^DC^ mice was abrogated (Fig. 2C). This result is in agreement with our previous study that *Map3k7* ^DC^ mice failed to respond to 2, 4‐dinitrofluorobenzene (DNFB)‐induced contact hypersensitivity 31. These results strongly suggest that TAK1 deficiency in DCs has an immunosuppressive effect in mice on both humoral and cellular immune function.

**Figure 6:**
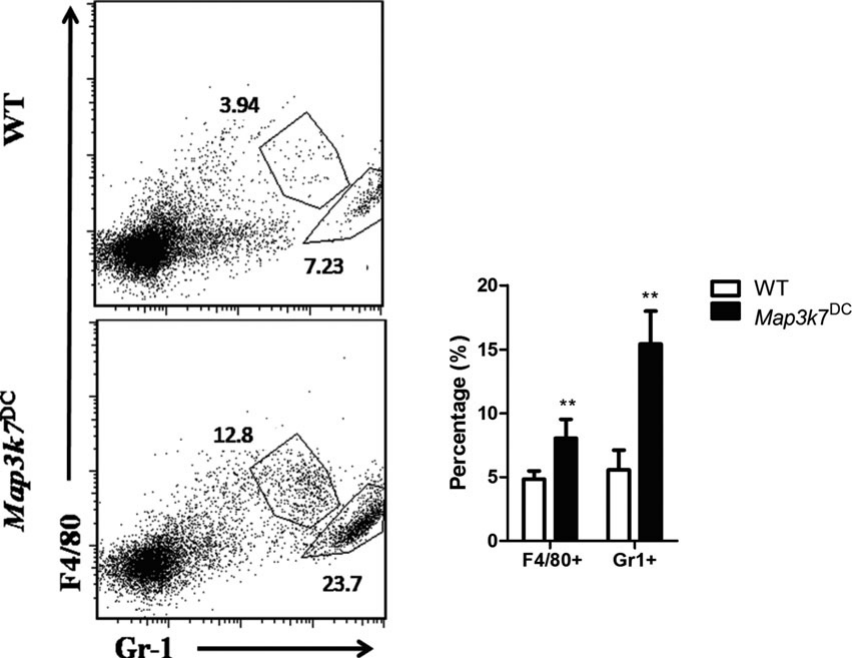
DC‐specific deficiency of TAK1 expanded macrophages and granulocytes. WT and *Map3k7* ^DC^ mice were immunized with SRBC as stated in the PFC assay section and the splenocytes were subtyped by flow cytometer. (Left) Flow cytometry analysis of splenic macrophages and granulocytes. (Right) The percentage of splenic cell subtypes. The bars represented mean ± SD (*n* = 5). **P < 0.01 indicate significant changes compared to the WT group.

To further clarify the mechanisms that the deletion of TAK1 in DCs caused immunosuppression, total, helper, and cytotoxic T‐cell percentages and counts, as well as total B‐cell and CD11c^+^ DCs percentages and counts in the spleens were analyzed via flow cytometry. It was clear that TAK1 deficiency in DCs decreased the percentage of total T cells, Th cells, Tc cells, and cDCs, but the percentage of B cells remained intact (Fig. 3C). Owing to the decreased total spleen cell number (Fig. 3A), the cell counts of all subsets of the immune cells above were markedly reduced in *Map3k7* ^DC^ mice compared with those in WT controls (Fig. 3D). In nonimmunized *Map3k7* ^DC^ mice, it also exhibited reduced populations of CD4^+^ and CD8^+^ T cells and increased apoptosis of cDCs in spleen 12. Collectively, TAK1 in DCs is essential for the homeostasis of both APCs and T‐cell populations, but has no impact on B cells. SRBCs are nonreplicating T‐cell dependent antigen that directly reach the spleen and induce the primary immune response there 32. DCs and T cells both play a key role in T‐dependent antibody formation. After T‐cell dependent antigen exposure, DCs uptake the antigen and subsequently process and present it to activate antigen‐specific Th cells. Then, Th cells activate B cells during cell–cell interaction and they also secrete several cytokines that induce B cells to proliferate and differentiate into plasma cells to produce antigen‐specific antibodies 33, 34, 35. Although *Map3k7* ^DC^ mice have intact splenic B cells proportion, they are lack of splenic cDCs and T cells to initiate the SRBC‐induced humoral immune response. On the other hand, the number of B cells in the spleen of *Map3k7* ^DC^ mice was much less than that in WT mice (Fig. 3D), which means that much less B cells could function in the immune responses. Besides, *Map3k7* ^DC^ mice showed significantly increased percentages of macrophages and granulocytes in the spleen (Fig. 6). As DCs were much decreased in the spleen, macrophages, another kind of APCs, may be recruited to the spleen to function as the main source of APCs or to remove the cellular debris that accumulates after TAK1 was knockout in DCs. In nonimmunized *Map3k7* ^DC^ mice, it also exhibited expansion of granulocytes resulting from myeloid proliferation as a consequence of loss of TAK1 in DCs 12. These might be possible explanations to why DC‐specific TAK1‐deficient mice are under immunosuppressed state.

Antigen‐primed CD4^+^ T cells can be divided into at least two subsets based on the different cytokine they produced and these subsets are known as Th1 and Th2. Th1 cells secrete IFN‐γ and IL‐2, which stimulates the maturation of DCs and improve the cellular immunity, and its transcription is depending on T‐bet 16. Th2 cells secrete IL‐4, IL‐5, IL‐6, and IL‐10 and its transcription factor is GATA3, which regulate the humoral immune response by inducing the proliferation and differentiation of B cells 36. The intracellular cytokine staining results exhibited that TAK1 deficiency in DCs abolished the Th1 and Th2 cytokines secretion by T cells in the spleen (Fig. 4). Along with this result, the real‐time PCR results showed that DC‐specific TAK1 deficiency significantly down‐regulated the mRNA expression levels of Th1 and Th2 cytokines (Fig. 5A). Besides, the expression of transcription factors T‐bet and GATA‐3, the master regulators controlling Th1 and Th2 cells differentiation, were also down‐regulated in the spleen of *Map3k7* ^DC^ mice (Fig. 5B), which indicated that deletion of TAK1 in DCs might inhibit the cytokines secretion by interfering with specific molecular programs to suppress Th‐cell differentiation. In nonimmunized *Map3k7* ^DC^ mice, the splenic T cells were impaired to produce IFN‐γ and IL‐2 upon acute polyclonal stimulation, indicative of altered function of T cells owing to TAK1 deletion in DCs 12. In agreement with the phenotype of other mice harboring myeloid proliferation syndrome 37, the ability of T cells to produce IFN‐γ was reduced. The impaired cytokine secretion function of T cells in spleen of *Map3k7* ^DC^ mice might attribute to the myeloid proliferation phenotype. And this further resulted in suppressing the SRBC‐induced humoral immune response.

MHC molecules play a crucial role in the response of T cells to antigens, which known as antigen processing and presentation 16. Our results showed that *Map3k7* ^DC^ mice significantly down‐regulated mRNA expression levels of MHC I/II molecules (Fig. 5D), indicative the diminished ability of APCs to activate T cells after immunized with SRBC. Chemokines and their receptors constitute a molecular system to regulate immune cell migration for effective and robust immunity, while CCR7 and CXCR4 are two common chemokine receptors 16. In this study, *Map3k7* ^DC^ mice showed significantly down‐regulated mRNA expression levels of CCR7 after immunized with SRBC (Fig. 5C). It is evident that CCR7 axis in the T‐ and B‐cell interactions is required to initiate effective high affinity humoral immune responses 38. The data revealed that the deletion of TAK1 in DCs disrupted normal function of CCR7 to induce the humoral immune response after immunized with SRBC. Taken together, TAK1 specific deficiency in DCs might damage the key molecules in the initiation of SRBC‐induced humoral immune responses.

In conclusion, our study demonstrated that TAK1 is critical for the development of humoral immune responses. Mechanistically, TAK1 is essential for survival of DCs, as well as the normal function and homeostasis of T‐cell populations. Therefore, DC‐specific targeting of TAK1 may be a feasible immunotherapy strategy.

## Author contribution

YP and ZL conceived and designed the study and conducted the experiments. XW and WH contributed to the interpretation of results and the discussion. YP wrote the manuscript. All authors have read and approved the final version submitted for publication.
